# Genome-Wide Scoring of Positive and Negative Epistasis through Decomposition of Quantitative Genetic Interaction Fitness Matrices

**DOI:** 10.1371/journal.pone.0011611

**Published:** 2010-07-15

**Authors:** Ville-Pekka Eronen, Rolf O. Lindén, Anna Lindroos, Mirella Kanerva, Tero Aittokallio

**Affiliations:** 1 Biomathematics Research Group, Department of Mathematics, University of Turku, Turku, Finland; 2 Data Mining and Modeling Group, Turku Centre for Biotechnology, University of Turku, Turku, Finland; 3 Division of Genetics and Physiology, Department of Biology, University of Turku, Turku, Finland; University College London, United Kingdom

## Abstract

Recent technological developments in genetic screening approaches have offered the means to start exploring quantitative genotype-phenotype relationships on a large-scale. What remains unclear is the extent to which the quantitative genetic interaction datasets can distinguish the broad spectrum of interaction classes, as compared to existing information on mutation pairs associated with both positive and negative interactions, and whether the scoring of varying degrees of such epistatic effects could be improved by computational means. To address these questions, we introduce here a computational approach for improving the quantitative discrimination power encoded in the genetic interaction screening data. Our matrix approximation model decomposes the original double-mutant fitness matrix into separate components, representing variability across the array and query mutants, which can be utilized for estimating and correcting the single-mutant fitness effects, respectively. When applied to three large-scale quantitative interaction datasets in yeast, we could improve the accuracy of scoring various interaction classes beyond that obtained with the original fitness data, especially in synthetic genetic array (SGA) and in genetic interaction mapping (GIM) datasets. In addition to the known pairs of interactions used in the evaluation of the computational approach, a number of novel interaction pairs were also predicted, along with underlying biological mechanisms, which remained undetected by the original datasets. It was shown that the optimal choice of the scoring function depends heavily on the screening approach and on the interaction class under analysis. Moreover, a simple preprocessing of the fitness matrix could further enhance the discrimination power of the epistatic miniarray profiling (E-MAP) dataset. These systematic evaluation results provide in-depth information on the optimal analysis of the future, large-scale screening experiments. In general, the modeling framework, enabling accurate identification and classification of genetic interactions, provides a solid basis for completing and mining the genetic interaction networks in yeast and other organisms.

## Introduction

Systematic screening of the phenotypic effects of combining pairs of mutations, relative to those of the single mutations, provides detailed information on the structure and function of genetic interaction networks [1]. Synthetic lethality is an extreme case of such genetic (or epistatic) interactions, in which pairs of single gene deletions that cause only a minor change in the phenotype individually are lethal in combination. Synthetic lethal interactions can reflect, for instance, non-essential elements of compensatory pathways that converge on the same essential endpoint function. Large-scale screening approaches for synthetic lethal interactions, such as those based on synthetic genetic arrays (SGA) or the diploid synthetic lethality analysis by microarray (dSLAM), have successfully been used in the past to map synthetic lethal interaction networks in model organisms such as yeast [2], [3]. These system-level maps have greatly improved our understanding of how mutations in different genes interact with one another to produce synthetic lethal or sick phenotypes [4]–[7]. Beyond such rather limited spectrum of aggravating epistatic effects (referred here generally to as ‘negative interactions’), the recent advances in the screening approaches have offered the means to start distinguishing a much broader range of quantitative phenotypes associated with pairs of mutations, including also alleviating epistatic effects (referred here to as ‘positive interactions’). In particular, high-throughput screening approaches, such as epistatic miniarray profiling (E-MAP) and genetic interaction mapping (GIM), have enabled systematic means to explore quantitative genotype-phenotype relationships on a large-scale [8]–[11]. Recently, the SGA approach has also been extended to allow unbiased, genome-wide mapping of quantitative genetic interaction networks [12]. These large-scale genetic interaction screening efforts are providing a new understanding of how genes function as networks to regulate cellular processes, either by enhancement or suppression, holding much promise for addressing many fundamental questions, such as buffering of genetic variation and evolution of complex diseases [13]–[16].

Despite the novel biological findings inferred from the large-scale genetic interaction experiments, there is no general consensus on how the massive datasets from these screens should be treated and analyzed. A wide range of statistical and computational strategies have been developed for modeling, mining, predicting and interpreting binary synthetic lethal/sick genetic interactions with other interaction information [17]–[29], yet only a limited effort has been devoted to developing generic analytic methodology for the needs of the large-scale quantitative genetic interaction experiments. Instead, custom-designed data handling pipelines have been tailored for the different screening approaches [10], [12], [30]–[32]; in particular, for the experimental design, customized processing and scoring, as well as downstream analysis of data from the E-MAP approach [33]–[37]. Compared to the heavily processed and scored E-MAP interaction matrices, the original double-mutant fitness measurements provided by the SGA and GIM analyses may encode more in-depth and elemental information on the complex genotype-phenotype relationships, but these datasets pose also modeling challenges beyond the reach of the traditional modeling strategies. In addition to asymmetric experimental design matrices, the modeling framework should cope, for instance, with, non-normal fitness value distributions and non-random missing value patterns. The high-dimensionality of the datasets poses also challenging computational problems; for instance, the current version of the SGA genetic interaction database comprises millions of quantitative fitness measurements [32]. The large number of mutant pairs screened, together with a relatively high experimental variability, can make it difficult to extract subtle interaction patterns from the background variability without a sound analytical framework and efficient algorithms [38]. Principled data modeling and mining strategies are therefore required not only to make the best use of the emerging quantitative interaction data, but also to evaluate the relative merits and potential limitations of the current large-scale interaction datasets; in particular, in terms of how well these enable scoring of both positive and negative classes of genetic interactions.

Classification of the quantitative genetic interactions is typically based on the concept of ‘expected fitness’. For instance, negative interactions, such as synthetic lethal and sick pairs, are inferred when a double mutation exhibits a more severe phenotypic effect than expected [1], [39]. Similarly, positive interactions are inferred when a double mutant phenotype is less severe than expected; these alleviating interactions can further be divided into categories such as suppression and masking, on the basis of the phenotype of the single mutants [1], [39]. Even more fine-grained sub-classifications can be made by further comparing the phenotypes of double and single-mutant strains to that of the wild type [40]–[41]. When applying such classification schemes one needs to first specify how the expected (or neutral) phenotype is defined under the null hypothesis that the strain carries two non-interacting mutations. It has been suggested that the multiplicative null model, based on the product of the two single-mutant fitness effects, provides an appropriate definition of genetic interactions in terms of being most accurate at identifying functional relationships [42]. These evaluations have often been made on high-resolution screens among a small set of genes related to a specific cell function [43], or among a set of known deleterious mutations causing significant growth defects [44]. Further, using simulated fitness data from a flux-balance analysis (FBA) model, a modified version of the product-based score, named ‘scaled epistasis’, has been introduced to provide better discrimination power, especially for the positive interaction pairs [45]. However, what remains unclear is the relative performance of the classification and scoring schemes on unbiased, large-scale, genetic interaction screens. Such evaluations are hampered by the lack of the single-mutant fitness measurements, which are rarely being available from the high-throughput interaction screens, but could be estimated in quantitative terms using computational modeling and the observation that both alleviating and aggravating epistatic events are relatively rare among a sufficiently large number of mutants that are, by and large, unrelated [1], [42].

Our aim here was to investigate the extent to which the currently available large-scale quantitative interaction datasets can capture the broad spectrum of epistatic effects, as compared to existing information on both positive and negative interaction classes, and whether the discrimination of the different interaction classes from the background variability could be improved by computational means. Our generic data transformation procedure is built on a decomposition model for the double-mutant fitness matrix. We have previously shown, using a high-resolution screen of genetic interactions among a small number of genes, that a rank-one matrix approximation can provide accurate estimates of the single-mutant fitness effects and improved prediction of functional relationships among the genes [46]. In the present study, we investigated whether this modeling strategy could be extended to the large-scale quantitative interaction screens to enhance the discrimination power of the original double mutant fitness matrix, using high-dimensional datasets from SGA, GIM and E-MAP screening approaches as example datasets. In contrast to previous works that have focused mainly on the negative interaction classes, we paid particular attention to the scoring of pairs of positive interactions, the accurate detection of which has motivated the development of these high-throughput quantitative screening approaches. By taking advantage of the extensive coverage of the large-scale datasets, we could assess the detection accuracy directly against the current knowledge of genetic interactions, extracted from independent studies, instead of using indirect evaluation criteria, such as functional relationships or physical interactions between the genes or their protein products. In addition to demonstrating that the matrix approximation can improve the detection of the various classes of genetic interactions beyond that obtained with the original datasets, we provide also systematic information about the optimal data transformation options and scoring functions for the different interaction classes and screening approaches, as well as novel predictions of positive interactions that remained undetected in the original SGA dataset.

## Results

The matrix approximation approach is based on the concept that most gene pairs in the large-scale genetic interaction screens have no significant interaction with each other, suggesting that the double-mutant fitness matrix **W** alone should carry enough information for the estimation of the vector **w** of single-mutant fitness effects under an appropriate null model (see Methods for details). After estimation of **w**, the interaction class of a mutant pair (*a*,*b*) was determined using specific scoring functions *S*, which transform the fitness matrix into a score matrix 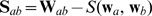. To demonstrate the effectiveness of this conceptual framework in analyzing high-dimensional data from the quantitative genetic interaction screens, we used three recent interaction datasets to systematically evaluate the information content of **S**, relative to that of **W**, with respect to the existing information on both positive and negative interaction classes as available in the BioGRID database (Table 1). The phenotypic suppression (PS) category is composed of the known positive interaction pairs (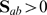), whereas the phenotypic enhancement (PE), synthetic sick (SS), and synthetic lethal (SL) categories represent with pairs of increasing degrees of negative interactions (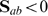), with SL being the extreme case (see Methods for details).

**Table 1:** The number of pairs of double mutations in the three datasets and the distribution of these pairs into the four BioGRID categories.

Dataset [reference]	Double mutants	Missing percentage	Phenotypic Suppression	Phenotypic Enhancement	Synthetic Sick	Synthetic Lethality
SGA [12]	3556280	10.11%	2973 (0.08%)	9780 (0.28%)	4281 (0.12%)	4812 (0.14%)
GIM [10]	173043	6.76%	118 (0.07%)	305 (0.18%)	60 (0.03%)	75 (0.04%)
E-MAP [9]	546105	34.01%	6350 (1.16%)*	24159(4.42%)†	5126 (0.94%)‡	5495 (1.01%)

*5453 of the PS pairs in BioGRID (85.87%) were extracted from this E-MAP dataset.

† 22743 of the PE pairs in BioGRID (94.14%) were extracted from this E-MAP dataset.

‡ 2 of the SS pairs in BioGRID (0.04%) were extracted from this E-MAP dataset.

The relative performance of the matrix decomposition-based data transformation procedure in distinguishing the four genetic interaction classes is demonstrated first using the unique data from the near whole-genome SGA screening effort, which is by far the largest quantitative genetic interaction dataset to date. At the time of the present analysis, the interactions extracted from this dataset were not yet stored in the BioGRID database, making the evaluation unsupervised in the sense that the information on the interaction classes is totally independent of the data used in their detection. The advantages of the data transformation procedure are further confirmed using two published large-scale quantitative interaction datasets; one obtained with the GIM screening approach and the other with the E-MAP approach (Table 1). The full detection results on each of the three datasets are provided in the supplementary Figures S1, S2, and S3, whereas the following sections focus mainly on demonstrating how our quantile-based matrix approximation (QMA) can help us to distinguish especially the pairs of positive interactions in the SGA dataset, compared to that of the alternating robust fitting (ARF), as well as on highlighting the added value of the scoring function and of the data pre-processing on the GIM and E-MAP datasets, respectively.

### Detecting genetic interactions in the SGA dataset

**Figure 1:**
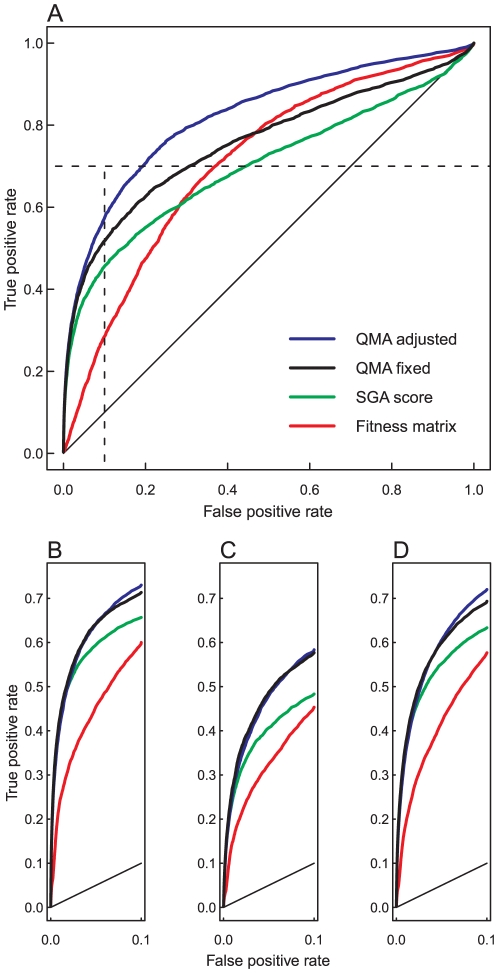
The detection of positive and negative genetic interactions in SGA dataset. True positive rate (TPR or sensitivity) is the fraction of gene pairs correctly classified into its true category, and false positive rate (FPR, or 1 - specificity) is the fraction of non-interacting gene pairs incorrectly classified into the particular category. (**A**) Classification performance in the phenotypic suppression (PS) category over the whole range of FPR (the overall AUC values are given in Table 2). The QMA method, together with the product scoring function, improved detection of this positive interaction class. For instance, at FPR of 10% (the dotted box), the original double-mutant fitness matrix identified 851 true PS interactions and the provided SGA score 1356, while QMA fixed (same parameters for all the categories) identified 1542, and QMA adjusted (specific parameters for the PS category) 1706 correct interactions (the sensitivity values at 10% FPR are given in Table 2). The lower insets depict the classification performance over the same specificity range in the three negative categories: (**B**) synthetic lethal, (**C**) synthetic sick, and (**D**) phenotypic enhancement. The minimum function was used in scoring the negative interaction classes.

The computational data transformation procedure improved the sensitivity and specificity of the detection of both positive and negative interaction categories in the SGA dataset, compared to that of using the provided double-mutant fitness matrix alone, or the SGA score matrix (Figure 1). While no further pre-processing was necessary in the SGA dataset, which was already normalized by its custom-designed computational procedures, the normalized dataset further benefited not only from the estimation of the single-mutant phenotypic effects using the fitness matrix approximation, but also from the ranking of the mutation pairs using scoring functions selected for the positive and negative categories separately. In particular, our QMA decomposition method was found out to be essential in the detection of positive interaction pairs (Figure 1A), whereas the alternative ARF matrix approximation method showed good performance in the negative interaction classes only (Figure S1). Interestingly, using the minimum of the two corresponding single-mutant fitness estimates as a scoring function provided optimal ranking performance in the detection of the negative interaction pairs, instead of the conventionally used multiplicative model, suggesting that the scoring function should be chosen separately for the negative and positive interactions.

As expected, adjusting the QMA parameters particularly to the positive interactions could further improve the detection of the PS category, especially at the higher levels of the false positive rate (FPR). The custom-designed SGA score showed its best performance at the lower levels of FPR, where the sensitivity of the double-mutant fitness matrix was relatively modest (Table 2). However, when considering the whole range of specificity, the original fitness matrix showed relatively good detection power, especially in the negative interaction categories (see the partial and overall AUC values in Table 2). While the QMA-based scoring improved systematically the detection of each interaction category, the biggest benefits were gained at the higher specificity levels, which are more relevant in many applications (see the 10% FPR window in the Figure 1). However, the superior early recognition performance did not come at the cost of missing many true interactions later on, as indicated by the higher sensitivity and specificity values in Table 2. In the identification of the most distinctive negative pairs, the QMA estimation parameters shared by both the positive and negative categories performed relatively well, compared to the parameters adjusted specifically to the three negative categories (Figures 1B–D), indicating that the matrix approximation method can be made relatively robust using the fixed mode.

**Table 2:** The detection accuracies of the four interaction categories in the SGA dataset before and after applying the QMA method.

Method/Category*	Sensitivity^a^	Specificity^b^	Partial AUC^c^	Overall AUC^d^
Phenotypic Suppression				
Fitness matrix	0.28624	0.63021	0.50597	0.70926
SGA score	0.45610	0.54753	0.55464	0.69713
QMA fixed†	0.51867	0.69354	0.62633	0.75706
QMA adjusted‡	0.57383	0.80663	0.70035	0.82182
Phenotypic Enhancement				
Fitness matrix	0.57536	0.83316	0.73539	0.85539
SGA score	0.63282	0.80444	0.69500	0.79130
QMA fixed	0.69131	0.89297	0.75943	0.84698
QMA adjusted	0.71902	0.91212	0.79286	0.87621
Synthetic Sick				
Fitness matrix	0.45106	0.71927	0.61399	0.76075
SGA score	0.48213	0.57533	0.57782	0.71689
QMA fixed	0.57487	0.68581	0.64313	0.77061
QMA adjusted	0.58141	0.74792	0.65914	0.78181
Synthetic Lethality				
Fitness matrix	0.59746	0.83714	0.73959	0.85550
SGA score	0.65628	0.83809	0.71764	0.80760
QMA fixed	0.71135	0.91072	0.76840	0.85026
QMA adjusted	0.72818	0.92201	0.79283	0.87472

*The minimum scoring function was used except for the PS category.

† The product scoring function was used for the PS category, and fixed QMA parameters for all the four categories.

‡ The product scoring function was used for the PS category, and adjusted QMA parameters to the PS category only.

a True positive rate (TPR, or sensitivity) at 10% false positive rate (FPR).

b 1-FPR (or specificity) at 70% TPR.

c Area under the curve (AUC) at 50% FPR.

d AUC at 100% FPR.

### Detecting genetic interactions in the GIM dataset

To confirm the good performance of the data transformation observed in the SGA dataset, we next evaluated its relative merits in another ‘non-zero-centered’ dataset measured with the GIM screening approach. Although this dataset was already publicly available, at the time of the analysis, there were no genetic interactions from this data in the four BioGRID interaction categories under study (Table 1), making the evaluation unbiased in the sense that the information on both the positive and negative interaction classes could be considered independent of the data used in their prediction. The computational data transformation procedure could again improve the detection of the various interaction classes in the GIM dataset, compared to its original double-mutant fitness measurements (Table 3). While the improvements in the positive PS category were not here as marked as in the larger SGA dataset, the negative interaction categories could be detected with very high accuracies after the data transformation. For instance, all the known SL pairs present in the GIM data matrix could be detected already at 50% FPR, when adjusting the QMA-parameters to the shared properties of the three negative interactions categories (Figure S2).

**Table 3:** The detection accuracies of the four interaction categories in the GIM dataset before and after applying the QMA method.

Method/Category*	Sensitivity^a^	Specificity^b^	Partial AUC^c^	Overall AUC^d^
Phenotypic Suppression				
Fitness matrix	0.42373	0.57654	0.55556	0.69060
QMA fixed†	0.45763	0.53825	0.56295	0.69890
QMA adjusted‡	0.52542	0.68653	0.62631	0.76629
Phenotypic Enhancement				
Fitness matrix	0.64918	0.82024	0.71584	0.80609
QMA fixed	0.71803	0.91352	0.77167	0.85240
QMA adjusted	0.72131	0.91560	0.80454	0.88562
Synthetic Sick				
Fitness matrix	0.65000	0.77092	0.69678	0.78482
QMA fixed	0.65000	0.80577	0.70115	0.78781
QMA adjusted	0.70000	0.88776	0.71100	0.80245
Synthetic Lethality				
Fitness matrix	0.76000	0.93597	0.77827	0.84940
QMA fixed	0.80000	0.96104	0.83869	0.91012
QMA adjusted	0.82667	0.96820	0.88909	0.94454

*The scaled epistasis scoring function was used except for the PS category.

† The minimum scoring function was used for the PS category, and fixed QMA parameters for all the four categories.

‡ The maximum scoring function was used for the PS category, and adjusted QMA parameters to the PS category only.

a True positive rate (TPR, or sensitivity) at 10% false positive rate (FPR).

b 1-FPR (or specificity) at 70% TPR.

c Area under the curve (AUC) at 50% FPR.

d AUC at 100% FPR.

**Figure 2:**
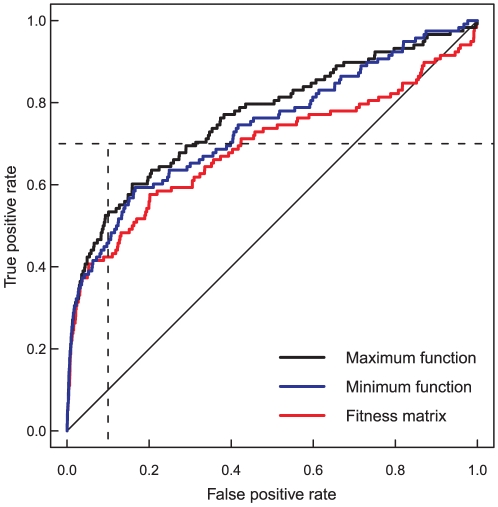
The effect of scoring function on detection of positive interactions in GIM dataset. True positive rate (TPR or sensitivity) is the fraction of gene pairs correctly classified into the phenotypic suppression (PS) category, and false positive rate (FPR, or 1 - specificity) is the fraction of non-interacting gene pairs incorrectly classified into the PS category. Compared to the minimum function, the maximum scoring function increased the AUC value from 0.732 to 0.766. The original double-mutant fitness matrix gave AUC of 0.690 (Table 3). In this illustration, the parameters of the QMA method were adjusted to the positive PS category individually (QMA adjusted).

Similar to the SGA dataset, data preprocessing did not improve the detection accuracy of any of the interaction categories in the GIM dataset. Interestingly, however, the scaled epistasis scoring function was found to perform better than the minimum or the product function in the GIM dataset, when detecting the negative interaction categories SL and PE (Table 3). Improving the detection of the SS category proved relatively challenging, regardless of the estimation method or scoring option; however, these results should be interpreted with caution due to the rather limited number of SS pairs in the GIM dataset (Table 1). Interestingly, we also observed that using the maximum of the two single-mutant fitness estimates, rather than their minimum, could further improve the detection of the positive PS pairs in this dataset (Figure 2). The maximum function has not been used before when scoring of quantitative genetic interactions, perhaps because the existing studies have focused mainly on negative genetic interactions. Our result suggests that the maximum definition may prove useful in scoring ‘extremely’ positive interaction pairs in large-scale genetic interaction experiments screened using GIM or similar quantitative screening approaches.

### Detecting genetic interactions in the E-MAP dataset

As a final evaluation data, we used the largest genetic interaction dataset available to date from the E-MAP screening approach. Although the customized interactions scores form this dataset have extensively been used in inferring genetic interactions in the BioGRID database, the stored pairs are mostly in the PE and PS categories (Table 1), making the evaluation results in the SL and SS categories unbiased. As expected, it was found out that our data transformation could not further improve the detection of the PE and PS categories in the E-MAP dataset (Table 4). However, it could effectively capture the properties of these categories even from this heavily processed and zero-centered fitness matrix, and provided accuracies almost as perfect as those obtained with the original interaction scores. In the E-MAP dataset, the minimum of the single-mutant fitness estimates provided the most appropriate scoring function in each category, supporting its good performance as a general option for defining interaction pairs in quantitative interaction datasets.

**Table 4:** The detection accuracies of the four interaction categories in the E-MAP dataset before and after applying the QMA method.

Method/Category*	Sensitivity^a^	Specificity^b^	Partial AUC^c^	Overall AUC^d^
Phenotypic Suppression†				
Fitness matrix	0.93354	0.99987	0.94920	0.96918
QMA fixed	0.91827	0.99349	0.93356	0.96038
QMA adjusted	0.93654	0.99680	0.94782	0.96896
Phenotypic Enhancement‡				
Fitness matrix	0.95016	0.99948	0.96096	0.97530
QMA fixed	0.95232	0.99924	0.95977	0.97508
QMA adjusted	0.87044	0.97047	0.91492	0.95288
Synthetic Sick				
Fitness matrix	0.50371	0.66926	0.60608	0.74443
QMA fixed	0.52204	0.73030	0.63454	0.77125
QMA adjusted	0.57121	0.83342	0.72329	0.84615
Synthetic Lethality				
Fitness matrix	0.71865	0.91313	0.77677	0.86101
QMA fixed	0.72757	0.91658	0.78968	0.87185
QMA adjusted	0.73722	0.91756	0.82527	0.90353

*The minimum scoring function was used is each category.

† The detection accuracy is highly overestimated since 85.87% of the PS pairs in the BioGRID were extracted from the E-MAP dataset.

‡ The detection accuracy is highly overestimated since 94.14% of the PS pairs in the BioGRID were extracted from the E-MAP dataset.

a True positive rate (TPR, or sensitivity) at 10% false positive rate (FPR).

b 1-FPR (or specificity) at 70% TPR.

c Area under the curve (AUC) at 50% FPR.

d AUC at 100% FPR.

**Figure 3:**
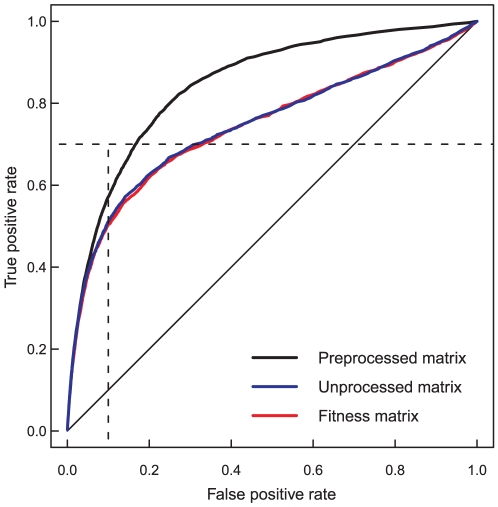
The effect of preprocessing on detection of synthetic sick pairs in E-MAP dataset. True positive rate (TPR or sensitivity) is the fraction of gene pairs correctly classified into the synthetic sick (SS) category, and false positive rate (FPR, or 1 - specificity) is the fraction of non-interacting gene pairs incorrectly classified into the SS category. Subtracting the row means of the provided fitness matrix increased the AUC value from 0.747 to 0.846. The original double-mutant interaction matrix gave AUC of 0.744 (Table 4). The QMA adjusted method together with the minimum scoring function was applied both to the original fitness matrix (un-processed) and its subtracted version (pre-processed).

In contrast to our expectations, however, the matrix approximation strategy could improve the detection of the negative SL and SS categories in the E-MAP data (Table 4). In particular, the gene pairs in the SS category were identified with relatively high accuracies using the adjusted QMA method. Due to this adjustment to the independent SL and SS categories, the detection of the PE category was somewhat compromised with the adjusted QMA, whereas the fixed version provided excellent performance in this category. More surprisingly, even though the E-MAP dataset has already been heavily processed with its custom-designed data analysis and scoring strategy, we found out that an additional preprocessing by simple subtraction of the row means of the original fitness matrix could markedly enhance the scoring of negative interaction categories such as SS (Figure 3). In contrast, the alternative ARF matrix approximation method performed poorer in the E-MAP dataset, regardless of whether or not the preprocessing option was used (Figure S3).

### Revealing novel positive interactions in the SGA dataset

**Figure 4:**
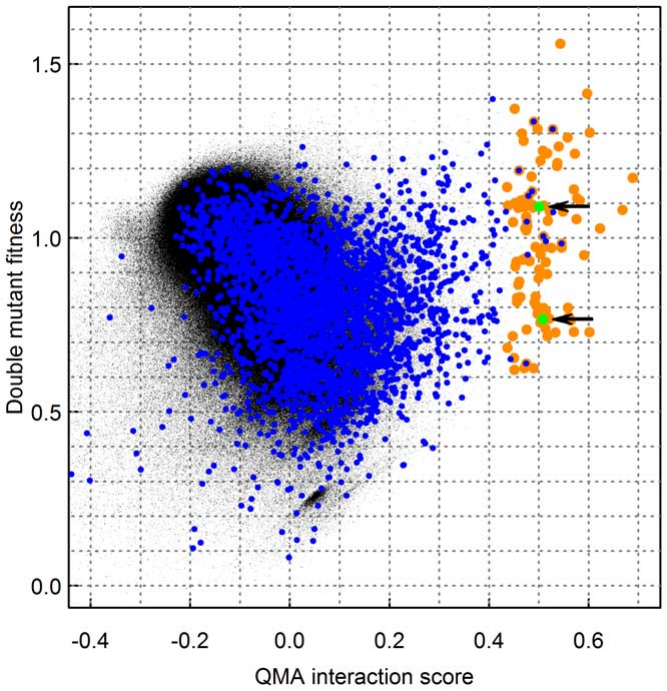
Examples of novel positive interaction pairs identified in the SGA dataset. Residual plot showing the association between the original double-mutant fitness values (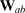, *y*-axis) and the interaction scores (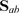, *x*-axis), as obtained using the QMA method with the product scoring function (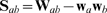). The large orange points indicate those 97 pairs that were among the 0.005 percent of the highest interaction scores, both in the fixed and adjusted version of the QMA, while the blue circles indicate those pairs that already belong to the positive interaction category of the BioGRID database (the 88 novel and 9 known positive pairs are listed in Table S1). The green points indicate the two interaction pairs, *nip100*Δs*ac1*Δ and *msc1*Δ*pat1*Δ, that are further discussed in the text (the arrows).

After having confirmed that the computational procedure can improve the detection of known pairs of genetic interactions in the quantitative interaction screening experiments, the obvious follow-up question is whether we can also identify such novel interacting pairs that are not yet reported in the BioGRID. As examples of such positive interaction pairs missed by the original SGA fitness data, we identified 88 new candidate pairs that were among the highest interaction scores in the transformed SGA dataset when using the product scoring function (Table S1), many of which would have remained unidentified using the provided fitness measurements alone (Figure 4). To offer robust and unbiased predictions for further analyses, we required that these pairs must be highly ranked not only with the parameters specific to the positive category (QMA adjusted), but also with the parameters shared by all the interaction categories (QMA fixed). Interestingly, the bulk of these pairs exhibited larger QMA scores than those known PS pairs already stored in the BioGRID database, indicating that these are not only novel but also plausible positive interactions. The overall pattern of association between the original and transformed fitness data illustrate that these two are, at least for the most part, complementary to each other (Figure 4).

Potential biological mechanisms were inferred for a number of these novel positive pairs, including *nip100*Δs*ac1*Δ, in which *nip100* (YPL174C) is a known component of the dynactin complex that is taking part, for instance, in chromatid separation during anaphase [47]. Other factors influencing the anaphase process include cytoskeletal components, such as astral microtubules and actin [48]. The gene product of its partner, s*ac1* (YKL212W), phosphatidylinositol phosphate phosphatase, influences, for instance, protein trafficing and actin cytoskeleton [49]. It has also been shown that *sac1*Δ, together with a deletion of an essential gene *ypp1* (YGR198W), restores cell viability [50]. Since both *nip100* and *ypp1* have a role in chromatid separation, these may also share a common positive interaction effect associated with a *sac1* mutant. In another pair, *msc1*Δ*pat1*Δ, it is known that an *msc1* (YML128C) mutant is defective in directing of recombination to homologous chromatids [51]. On the other hand, *pat1* (YCR077C) is necessary for accurate chromosome segregation during mitosis and meiosis [52]. Thus, it is possible that the problems caused by the unequal sister-chromatid recombination could be alleviated by the inaccuracy in the segregation of chromosomes during meiosis, thus explaining why the double-mutant may become more viable compared to the single-mutants.

## Discussion

The present study demonstrated that the double-mutant fitness matrices, obtained from the large-scale quantitative interaction screens, alone contain sufficient information according to which it is possible to distinguish gene pairs encoding both positive and negative classes of interactions. The matrix decomposition-based computational procedure, which effectively estimates and corrects the single-mutant fitness effects, was able to further improve the detection accuracy of each interaction category, especially in those datasets, such as SGA and GIM, in which the original fitness measurements were provided. Both the SGA and GIM datasets captured relatively well the known interaction pairs, as extracted from the BioGRID database, especially the negative interactions, whereas the detection of the positive interactions proved much more challenging when using the provided double-mutant fitness matrix alone. The matrix decomposition strategy, combined with the selected scoring functions, transformed the original fitness matrix so that it allows better discrimination of the various negative or positive interaction classes from the background variability. Accordingly, we could identify the strongest interaction pairs in each category much earlier in the transformed score matrix than in the original fitness matrix, or using the custom-designed scoring scheme in the SGA dataset (Figure 1). The computational procedure can therefore be used, for instance, in prioritization of the most promising interactions for follow-up functional analyses or more targeted screening experiments among the exponential number of combinations.

Our generic data transformation procedure is streamlined for unbiased, explorative research. For instance, it can avoid performing any tedious filtering steps, but the provided screening data can be used as its input. In fact, the detection of interaction classes could be made with higher accuracies in the untreated SGA and GIM datasets than in their filtered or pre-processed versions. This suggests that it is neither necessary nor even recommendable to filter down the number of double-mutant measurements, prior to the actual downstream data analyses, since such filtering can lose important discoveries. The E-MAP dataset was already heavily pre-processed and custom-scored, as has been noted also in previous works [26], and therefore it was not expected that the matrix decomposition could provide marked improvements in this data. Surprisingly, however, the matrix approximation strategy could improve the detection of those categories of BioGRID that were independent of the E-MAP data under the analysis (SL and SS; see Table 4). This may be attributed to the simple pre-processing, which subtracts the slightly negative row means from the original E-MAP score matrix, and thereby transforms the preprocessed matrix closer to the original double-mutant fitness matrix. This pre-processing had a minor effect on the internal consistency of the E-MAP dataset, decreasing the correlation between the reciprocal pairs that were screened as both queries and arrays to 0.961. In general, these systematic results on the predictive power of the three high-throughput quantitative screening approaches (SGA, GIM and E-MAP) should prove useful in the design and analysis of the future screening experiments conducted using these or similar screening methodologies.

The combination of QMA parameters, pre-processing options, and scoring functions was specific both to the different interaction datasets as well as to the interaction classes under analysis (Table 5).

**Table 5:** The QMA parameters, pre-processing options, and scoring functions recommended for the different interaction datasets and classes.

Method/Dataset*	QMA parameters 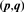	Pre-processing option	Positive interactions	Negative interactions
SGA				
Fixed	(0.55,0.95)	No	Product	Minimum
Adjusted P	(0.10,0.95)	No	Product	
Adjusted N	(0.95,0.50)	No		Minimum
GIM				
Fixed	(0.60,0.50)	No	Minimum	Scaled epistasis
Adjusted P	(0.05,0.95)	No	Maximum	
Adjusted N	(0.80,0.25)	No		Scaled epistasis
E-MAP				
Fixed	(0.50,0.60)	Row mean	Minimum	Minimum
Adjusted P	(0.30,0.65)	Row mean	Minimum	
Adjusted N	(0.50,0.15)	Row mean		Minimum

*The QMA parameters and other options were specific to the interaction datasets and classes, resulting in three combinations per dataset: one for scoring both positive and negative interactions (Fixed), and the others for scoring the positive and negative interactions separately (Adjusted P and N, respectively).

It is likely that the differences in the pre-processing options and QMA parameters can be mostly attributed to the technical differences in the screening approaches and their specific fitness readouts, whereas the observed differences in the scoring functions may be more closely linked to the underlying cellular mechanisms contributing to the biologically distinct classes of alleviating and aggravating interactions. Interestingly, it was found out that the conventional multiplicative product model was the optimal choice only when detecting positive interactions in the SGA dataset, whereas the minimum function performed generally better when scoring various types of interactions in the different datasets (Table 5). These observations raise many interesting follow-up questions for future experimental and computational studies. For instance, the maximum scoring function was found beneficial when detecting positive interactions in the GIM dataset (Table 5); however, in more general terms, there seems to be many subtypes of the alleviating interactions, the optimal separation of which may need more tailored screening and classification schemes. Moreover, even if the ‘scaled epistasis’ scoring function was found useful in certain negative interaction categories, it produced virtually unlimited interaction scores in some cases, thus necessitating its further improvements and modifications to make it numerically stable.

There are some potential limitations in the present evaluation setting. For one thing, the present detection accuracies may be severely underestimated by the fact that we were likely to detect a large number of such true genetic interactions that have not yet been stored in the BioGRID database (hence labeled as false positives), as well as such plastic interactions that have previously been identified under experimental conditions other than those used in the SGA or GIM experiments (hence labeled as false negatives). In the absence of an established set of known non-interacting mutant pairs, similar to that available for physically non-interacting protein pairs [53], we used here simply the complement of each category as the reference sets of neutral pairs. This definition should not favor the relative performance of any of the methods under comparison. As positive interaction class, we used the PS category of BioGRID, rather than synthetic rescue (SR), because the interactions in the SR category overlap heavily with negative categories and were often defined on the basis of triple mutations. In spite of such problems in defining the positive and neutral sets, we could already provide relatively high detection accuracies. For instance, the accuracies for the negative classes are comparable to those obtained with supervised machine learning classifiers, such as support vector machines or decision trees, which operate exclusively on the BioGRID SL/SS categories [27]–[29]. It can therefore be expected that even higher accuracies will be obtained when the interaction scores from our procedure are combined with the fully supervised approaches.

While substantial effort has been devoted to extracting and storing reliable genetic interaction data [30]–[32], [54]–[56], standards for the computational analysis are still lacking, perhaps because the choice of the modeling strategy depends both on the screening approach and on the goals of the experiment. The downstream analysis methods, such as data clustering or functional analyses [12], [38], are more targeted at addressing the biological questions under study, whereas the upstream data analysis tools, such as quality control and data normalization [31], [32], are aimed at correcting for the sources of experimental variation in the screening experiments to provide reliable fitness data for the analyses. Our objective here was to further transform the custom-normalized data through the matrix-based modeling framework, which offers a quantitative and flexible means to deal with the properties of the different assays, while avoiding over-fitting that would bias the downstream analysis objectives. The transformed data matrix can subsequently be used in the downstream data analysis phases, using either the individual interaction scores or the rows of the score matrix (i.e. genetic profiles). This computational work therefore complements the ongoing experimental efforts by providing a platform for mining the genetic interaction networks in yeast and other organisms. In-depth understanding of the quantitative relationships behind genetic interactions and their contributions to various phenotypes in model organisms may later translate into an improved identification of the genetic variation responsible for polygenic disorders that is beyond the capability of the current genome-wide association studies.

## Materials and Methods

**Figure 5:**
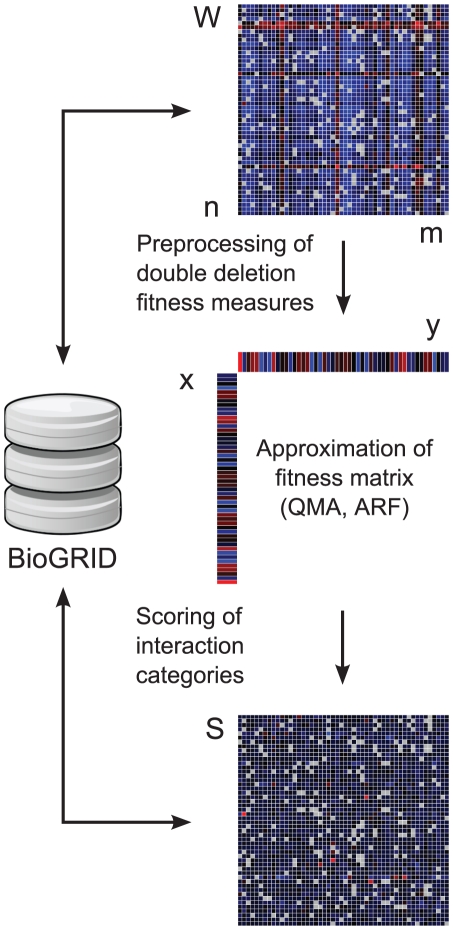
The schematic of the data transformation procedure. The input is the provided double-mutant fitness matrix **W**, with columns and rows corresponding to the *m* query and *n* array strains, respectively. The example data matrix here depicts a selected sub-matrix screened using the synthetic genetic array (SGA) technology (the blue and red matrix entries indicate positive and negative effects, respectively, whereas the grey elements are missing values). In the first step, the original fitness matrix **W**, or its pre-processed version, is decomposed using a rank-one approximation method, such as quantile-based matrix approximation (QMA) or alternating robust fitting (ARF), resulting in two vectors, **x** and **y**, which model the variability across the query and array mutants, respectively. Here, the array vector **x** is used as an estimate for the single-mutant fitness effects **w**, whereas the query vector **y** is used for correcting the technical variation between the screens. The individual gene pairs in **W** are then ranked according to their evidence for a genetic interaction, using specific scoring functions for positive and negative interaction classes. The output of the procedure is a score matrix **S**, which has the same dimensionality as the original data matrix **W**. The information content of both **W** and **S** are evaluated with respect to the interaction categories stored in the BioGRID database (version 2.0.51).

Figure 5 illustrates the computational procedure used for transforming a quantitative genetic interaction dataset and for evaluating its discrimination power before and after the transformation. The purpose of the data transformation is to improve the scoring of the various interaction classes using the double-mutant fitness measurements together with the single-mutant fitness estimates, obtained through the matrix approximation procedure. The following sub-sections detail the quantitative interaction datasets used in its evaluation phase, as well as the distinct steps of this generic computational procedure.

### Genetic interaction datasets

Three large-scale quantitative genetic interaction datasets on budding yeast (*Saccharomyces cerevisiae*), as available from different screening technologies, were used to demonstrate the performance of our computational data transformation procedure. By far the largest dataset at the time of the analysis was a pre-release version of the SGA screening experiment [12]. This massive screening effort is continuously increasing in coverage and it has recently been made publicly available via the DRYGIN (Data Repository of Yeast Genetic Interactions) database [32]. The pre-release version of the data matrix was based on a total of 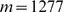 SGA screens, in which each mutant of interest (so-called ‘query strain’) was individually crossed to non-essential gene deletion collection (‘array strains’). More specifically, three types of query strains, namely 1091 non-essential gene deletions, 101 temperature-sensitive essential gene alleles and 85 hypomorphic DAmP (decreased abundance by mRNA perturbation) alleles, were crossed to each of the 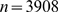 non-essential array strains. Colony sizes were measured for the pairwise double-mutant combinations in four replicates; of these measurements, 10% were filtered out for technical reasons (resulting in missing data points). The double-mutant fitness values provided by the SGA analysis were extensively corrected for several sources of systematic variation associated with the measurement process [32]. The filtered and normalized double-mutant fitness data matrix, denoted here by **W**_SGA_, was treated as the input of our computational data transformation pipeline (see Figure 5). In addition to the double-mutant fitness values, the SGA dataset includes a customized scoring scheme, which quantizes the extent to which a double mutant colony size deviates from the colony size expected from combining the two mutations together (referred here to as ‘SGA score’). In the evaluation results, this custom-designed SGA score was used as a reference for our interaction scoring strategy.

The two other genetic interaction datasets were available from published literature at the time of the analysis; one obtained with the GIM screening approach and the other with the E-MAP approach. The GIM dataset contains the quantitative growth measurements from its pilot experiment [10], which involves double-mutant fitness measurements among 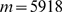 query gene mutations and 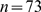 array gene mutations from the yeast deletion collection. Similar to the SGA approach, the double mutants were also generated by mating and sporulation but in a single pool combining all non-essential gene deletions of the collection. Similar to the dSLAM approach, the estimation of the individual double mutants' relative growth rates was performed by using bar-code DNA microarrays. For every screen, two experiments were run in parallel, one with the query gene deletion strain and another with a reference deletion. The normalized growth results were expressed as log-ratios between the query population and the reference population. Signal-to-noise ratio across technical replicates was used to filter out non-reproducible measurements (7% of the gene pairs). The filtered fitness effects were transformed back to non-log-scale to produce the **W**_GIM_ data matrix. The E-MAP dataset was available from the epistatic miniarray profiling study of quantitative genetic interactions between 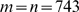 genes involved in various aspects of yeast chromosome biology [9]. The mutations included deletions of 663 non-essential genes and hypomorphic alleles for 70 essential genes, constructed using the DAmP strategy. In addition to relatively heavy filtering (34% missing rate), these screening data were already custom-processed and scored against an expected fitness [31], resulting in a symmetric and close to zero-centered data matrix **W**_E-MAP_.

### Data preprocessing options

After the custom-normalization and filtering out unreliable measurements, there may still remain sources of experimental variation that can confound the true phenotypic variation. As the first data transformation step for a given double-mutant fitness matrix **W** (Figure 5), we investigated whether some simple data preprocessing option, such as removing potential location shifts in **W** before performing its approximation, was able to improve the discrimination power of the data matrix. The presumption was that such a data preprocessing could potentially make the null model for the non-interacting genes more distinctive, and therefore facilitate its estimation by means of matrix-approximation methods, especially in those datasets, such as SGA and GIM, in which the original double-mutant fitness measurements were available. It was also hypothesized that in the custom-scored datasets, such as E-MAP, this kind of data pre-processing would provide only a marginal, if any, contribution to the overall performance of the data transformation procedure. More specifically, three different preprocessing options were evaluated for each of the data matrices individually: subtraction of the row or column means component-wise from the rows or columns of **W** separately, or subtraction of the grand mean calculated as the average of the values over all the entries in **W**.

### Matrix approximation methods

At the core of the data transformation procedure is the rank-one matrix approximation-based estimation of the single-mutant fitness effects **w** (Figure 5). The approximation of the double-mutant fitness matrix **W**, or its preprocessed version, was based on the observation that significant genetic interactions are rare and that the multiplicative model is a reasonable approximation in the case of no interaction [42], [46]. Accordingly, the double-mutant fitness matrix alone should carry enough information for accurate estimation of the single-mutant fitness values, which typically are not measured in the large-scale interaction experiments, but could be estimated computationally. The standard practice for calculating a low-rank matrix approximation is by means of the singular value decomposition (SVD). Formally, the SVD of a real 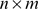 matrix **W** can be written as
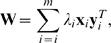
(1)where 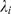 is the *i* ^th^ eigenvalue of **W**, and 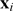 and 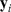 are the corresponding left and right eigenvectors, respectively. It is well known that if the summation is truncated to the first *k* terms, then the right hand side of Eqn. 1 is the least-squares rank-*k* approximation to **W** [57]. The special case of SVD with 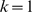 yields two components, **x** and **y**, where the *n*-dimensional array vector **x** models the within-screen variability and it is therefore used as an estimate of the single-mutant fitness vector **w**, while the *m*-dimensional query vector **y** models the between-screen variability and it can therefore be used for normalization purposes. The conventional computation of SVD requires that the matrix **W** is free of missing entries and outliers, which is rarely the case in the high-throughput interaction screens. Therefore, the estimation problem must in practice be solved by numerical means.

#### Sequential matrix approximation (SMA)

In case the dimensionality of the double-mutant fitness matrix **W** is moderate, the matrix approximation problem can be treated using iterative procedures, which solve the weighted least-squares optimization problem, in which binary weights can be employed to ignore the effects of missing entries [58]. If further the matrix **W** is symmetric, one can use a numeric estimate for **w** based on the eigenvector of the largest eigenvalue of **W** in its spectral decomposition (in the symmetric case, 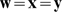). This symmetric strategy was used in our previous work [46], in which the SMA method was developed and applied to a small-scale, square (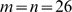) high-resolution genetic interaction data matrix [43]. To extend SMA strategy to cope with the large-scale genetic interaction datasets, we have further developed several of its components. For instance, SMA was modified to deal with unbalanced experimental designs, as well as differences between the screens, resulting in potentially highly rectangular, asymmetric matrices **W** [58], such as those provided with the SGA and GIM datasets. However, it was soon found out that the built-in sequential procedure, which considers such increasingly larger subsets of mutation pairs in **W** that best fit the multiplicative model to improve the estimation of the single-mutant fitness vector **w**, was computationally infeasible when faced with the massive data matrices originating from the high-throughput genetic interaction experiments. Therefore, even if the SMA showed good potential, this strategy was not used here in the systematic evaluation results.

#### Alternating robust fitting (ARF)

To reduce the computational complexity when scaling up to the high-dimensional screening data, we searched for more efficient rank-one approximation methods used in the context of high-throughput experiments. One candidate was ARF, a robust variant of SVD, previously applied to asymmetric data matrices from gene expression microarray experiments [59]. The method is based on alternating regression of the rows and columns of **W** in a sequential manner. Briefly, starting with an initial estimate for the unit column vector **y**, the matching scaling for the row vector **x** is obtained by fitting linear regression row-by-row using non-missing entries of **W**. In the next step, the estimated row coefficients **x** are taken as given, and the linear regression is used in exactly the same way to calculate new estimates of the column coefficients **y**. This process is continued until convergence or enough iteration steps have been performed. Instead of using the standard least-squares regression, which is sensitive to outliers (e.g. extreme fitness measurements), we used least trimmed squares regression similarly as in the original work [59]. As an initial estimate, we used the leading term of SVD of the matrix **W** with missing values substituted by ones. The ARF method includes two parameters: a trimming parameter *t*, which determines the percentage of the values used in fitting the regression model, and a Boolean parameter *a*, which indicates whether or not to include an intercept term in the regression model. While the ARF method performed well in the negative interaction categories, it could not distinguish the positive interaction pairs, even after adjusting its parameters separately to each interaction class (Figure S1).

#### Quantile-based matrix approximation (QMA)

In this work, we develop a novel and efficient rank-one matrix approximation method, named QMA, to address the problem of detecting accurately also the positive end of interactions, yet being simple enough for the large-data interaction datasets. The matrix approximation method is conceptually similar to the Tukey's median polish procedure [60], except that QMA uses multiplicative model instead of additive model, division in place of subtraction, arbitrary quantile points instead of fixed medians, and performs one iteration only rather than continuing until convergence or pre-defined number of iteration steps. More specifically, we obtain the estimate for **w** by calculating the *p*-quantile points separately for each of the rows in **W** and then arranging these quantile in the array vector **x**. In case there are negative entries in the matrix **W**, such as in the E-MAP dataset, then the (1-*p*)-quantile is used instead for those rows in which more than half of the components are negative. In the next phase, the rows of **W** are divided by the components of **x**, thus resulting in a new matrix 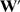. Finally, vector **y** is obtained similarly by calculating the *q*-quantiles for the columns in 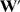. Such estimates **x** and **y** have the desired property that if **W** was originally a rank-one matrix, then the QMA method provides an exact approximation, that is, 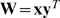, for any 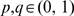. The two-way median estimate is a special case of QMA, with 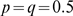. This simple two-phase matrix decomposition method is relatively fast, with computational complexity of the order of 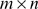, compared to the iterative or sequential alternatives. It can also deal effectively with the technical issues in the large-scale genetic interaction datasets, namely non-random missing value distribution (e.g. the filtered measurements), both positive and negative extreme observations (e.g. the synthetic lethal and suppression pairs), and fitness effects following non-normal distributions (e.g. in the SGA and GIM datasets). The R-codes for the QMA algorithm are available upon request from the authors.

### Scoring of the interaction classes

The final phase of the data transformation procedure involves scoring of each individual gene pair, say (*a*,*b*), on the basis of its double-mutant fitness measure 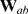 and the two single-mutant fitness estimates 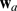 and 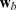, as obtained from the array vector **x** (Figure 5). The objective of such scoring was to decide whether or not the gene pair encodes a true genetic interaction. It became soon evident that any single scoring function could not provide optimal scoring capability for all the interaction datasets and interaction categories considered. Therefore, we systematically evaluated the performance of several scoring function alternatives, including those introduced in previous experimental and theoretical works [42], [45], as well as our own candidates for additional scoring functions. More specifically, we reported in the present work the results obtained with the following four scoring functions (referred to as ‘minimum’, ‘maximum’, ‘product’ and ‘scaled epistasis’):
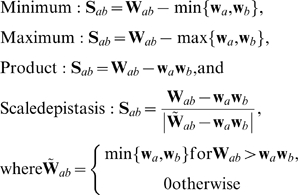
It was noted that the scaled epistasis function, as defined in the original work by Segre et al. [45], can produce virtually unlimited score values for some specific cases of the triple (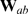, 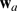, 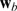), for instance, for such positive interaction pairs in which one of the single-mutant phenotypes is close to that of the wild type and the other single-mutant has a more severe phonotype than the double-mutant (*i.e.* 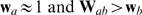). Therefore, its values were later truncated to 1000. However, such extreme score values were encountered only very rarely in the course of the evaluations, and therefore these had only a negligible effect on the results. The application of a scoring function individually to each of the gene pairs (*a*,*b*) transformed the double-mutant fitness matrix **W** into a score matrix **S**, which preserves the dimensionality of the original fitness matrix, but has model residuals as its entries instead of the double-mutant fitness measurements.

### The BioGRID interaction categories

In the evaluation phase, the non-missing entries of the matrices **W** and **S** were evaluated in terms of their information content for distinguishing both positive and negative interaction classes (Figure 5). As the ground truth for the genetic interaction pairs, we used the four different genetic interaction categories as available from the BioGRID database, version 2.0.51 [54]. This database contains genetic interactions extracted from both small-scale and high-throughput interaction screening studies. The interaction categories used in the present study were: Synthetic Lethality (SL), mutations in separate genes, each of which alone causes a minimal phenotype, but result in lethality when combined; Synthetic Growth Defect (or synthetic sick, SS), mutations in separate genes, each of which alone causes a minimal phenotype, result in a significant growth defect; Phenotypic Enhancement (PE), mutation or over-expression of one gene results in enhancement of any phenotype (other than lethality or growth defect) associated with mutation or over-expression of another gene; and Phenotypic Suppression (PS), mutation or over-expression of one gene results in suppression of any phenotype (other than lethality/growth defect) associated with mutation or over- expression of another gene. The PS category is composed of the positive interaction pairs (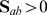), while the PE, SS, and SL categories include pairs with different degrees of negative interactions (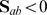).

### Evaluation of the detection accuracy

The detection accuracy of the data matrices **W** and **S** in distinguishing the four interaction categories (SL, SS, PE and PS) from the background variability (i.e. the complement of the category in the dataset under evaluation) was assessed using the receiver operating characteristic (ROC) curves [61]. Briefly, the ROC curve characterizes the relative trade-off between the true positive rate (TPR) and the false positive rate (FPR) of the data matrix over the range of possible discrimination thresholds for a selected scoring function. Here, TPR (or sensitivity) is the fraction of the gene pairs correctly classified into its true category, and FPR (or 1 - specificity) is the fraction of the non-interacting gene pairs incorrectly classified into the BioGRID category. The overall prediction performance was summarized using the area under the ROC curve (AUC). For an ideal classifier, TPR = 1, FPR = 0 and AUC = 1, whereas a random classifier has on average AUC of 0.5. To investigate the performance of a method at low FPR levels, we calculated the partial area under the curve up to a selected FPR threshold, and standardized it to have the maximum value of one [62]. In many applications, where the goal is to find a set of candidate interaction pairs for further conformational studies, only the gene pairs identified at low FPR levels are relevant. Therefore, in addition to the overall AUC levels, we evaluated the practical performance of the methods using different FPR and TPR thresholds.

### Implementation issues for the methods

The array and query vectors, **x** and **y**, from the QMA or ARF methods are unique up to scaling. To provide unique estimates of the single-mutant fitness vector **w**, we scaled the array vector **x** using the set of mutants shared by the array and query strains. More specifically, we defined that **w** equals to 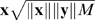, where *M* is the median of the non-negative ratios 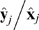 over those components *j* that belong both to the array and query arrays; here, 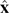 and 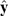 are rescaled to unit length, that is, 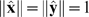. The ARF method gave sometimes estimates **x** and **y** with all elements negative. Therefore, in those case in which the median of **x** was negative, we multiplied both **x** and **y** by −1 (this does not affect the rank-one approximation 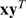, only the single-mutant fitness estimate **w**). Similar to the ARF methods, the two parameters of the QMA 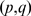 were adjusted individually to each of the three datasets, with three parameter combinations per dataset: one for scoring all the four categories (QMA fixed), and the others for scoring either the negative or positive categories separately (QMA adjusted). It should be noted that the parameter *p* determines the unit vector 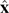, whereas the parameter *q* affects the length 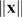 only. To guarantee that the array vector **x** could be used similarly in the estimation of each of the single-mutant fitness effects in **w**, the evaluations presented here were based on the sub-matrices of **W** and **S**, constructed by including only those columns in which the query mutant corresponds to one of the array mutants. This makes the estimation results more comparable at the cost of losing some the gene pairs in the evaluation phase.

## Supporting Information

Figure S1 The full ROC curves showing the detection accuracy of the different genetic interaction categories in the SGA dataset using the QMA and ARF methods. The four interaction categories are shown as separate panels, and the two methods as separate sets of ROC curves on the two pages.
(0.13 MB PDF)

Figure S2 The full ROC curves showing the detection accuracy of the different genetic interaction categories in the GIM dataset using the QMA and ARF methods. The four interaction categories are shown as separate panels, and the two methods as separate sets of ROC curves on the two pages.
(0.10 MB PDF)

Figure S3 The full ROC curves showing the detection accuracy of the different genetic interaction categories in the E-MAP dataset using the QMA and ARF methods. The four interaction categories are shown as separate panels, and the two methods as separate sets of ROC curves on the two pages.
(0.11 MB PDF)

Table S1 Candidate gene pairs showing evidence for positive genetic interactions as identified in the SGA dataset using the matrix decomposition strategy and the product scoring function (named ‘QMA score’). The nine ORF pairs already stored in the BioGRID-PS category are boldfaced.
(0.01 MB PDF)
