# Supplementary material for: Genome-Wide Scoring of Positive and Negative Epistasis through Decomposition of Quantitative Genetic Interaction Fitness Matrices
Source: PLoS One. 2010 Jul 15;5(7):e11611. doi: 10.1371/journal.pone.0011611 (PMC2904709; doi:10.1371/journal.pone.0011611)
Supplement: Figure S1 — The full ROC curves showing the detection accuracy of the different genetic interaction categories in the SGA dataset using the QMA and ARF methods. The four interaction categories are shown as separate panels, and the two methods as separate sets of ROC curves on the two pages. (0.13 MB PDF) [file pone.0011611.s001.pdf]

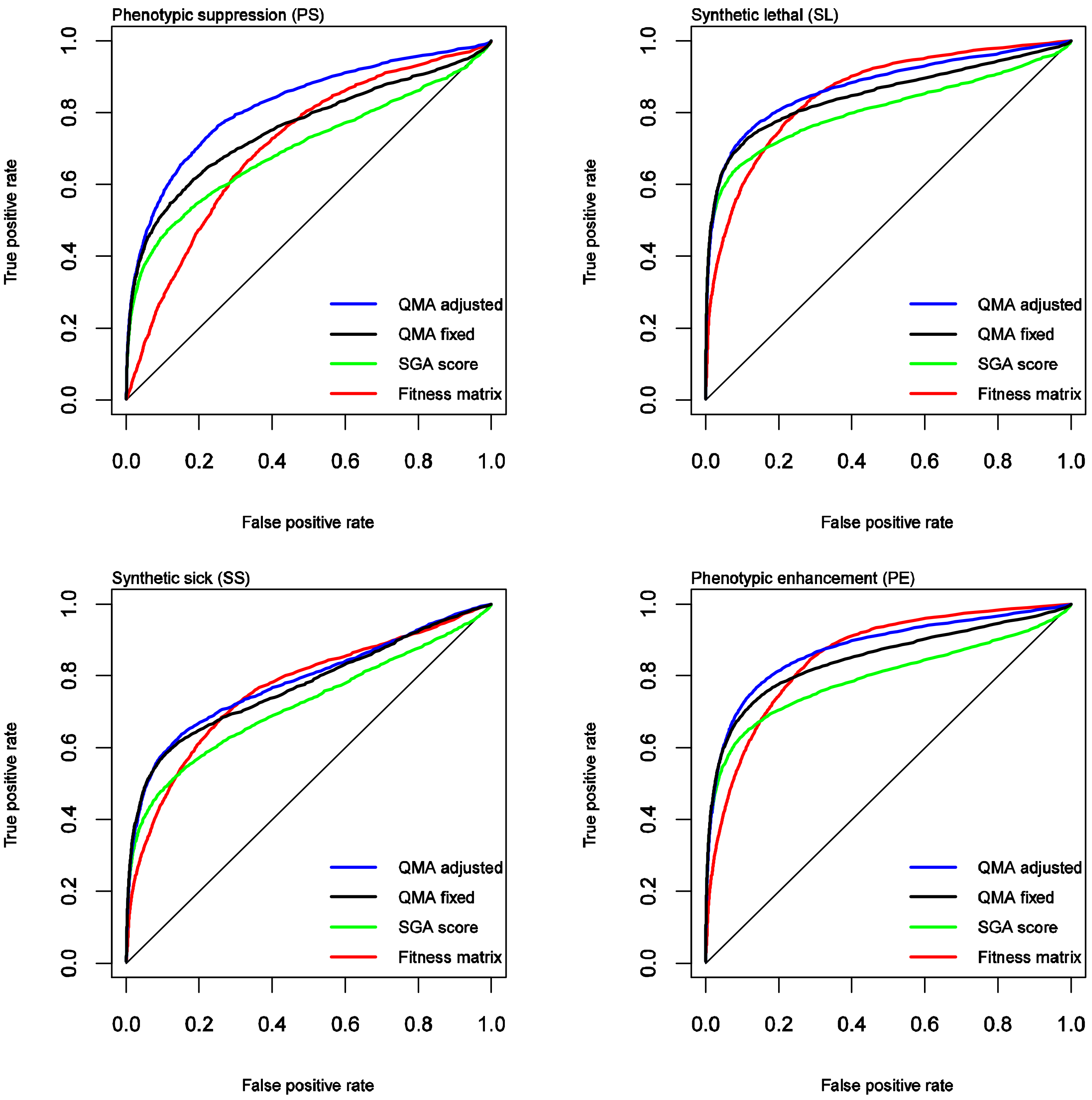

SGA-dataset: The QMA parameters fixed to the four interaction classes were p = 0.55, q = 0.95 (black curve). The QMA parameters adjusted to the positive (PS) and negative classes (SL, SS, and PE) were p = 0.10, q = 0.95 and p = 0.95, q = 0.50, respectively (blue curve). The original fitness measurements (red curve) and the customized SGA interaction scores (green curve) are shown as references. The product function was used for scoring the positive interactions and the minimum function for scoring the negative interactions. The values of the original double-mutant fitness matrix were traversed in reverse order when performing ROC-analyses in the PS category.

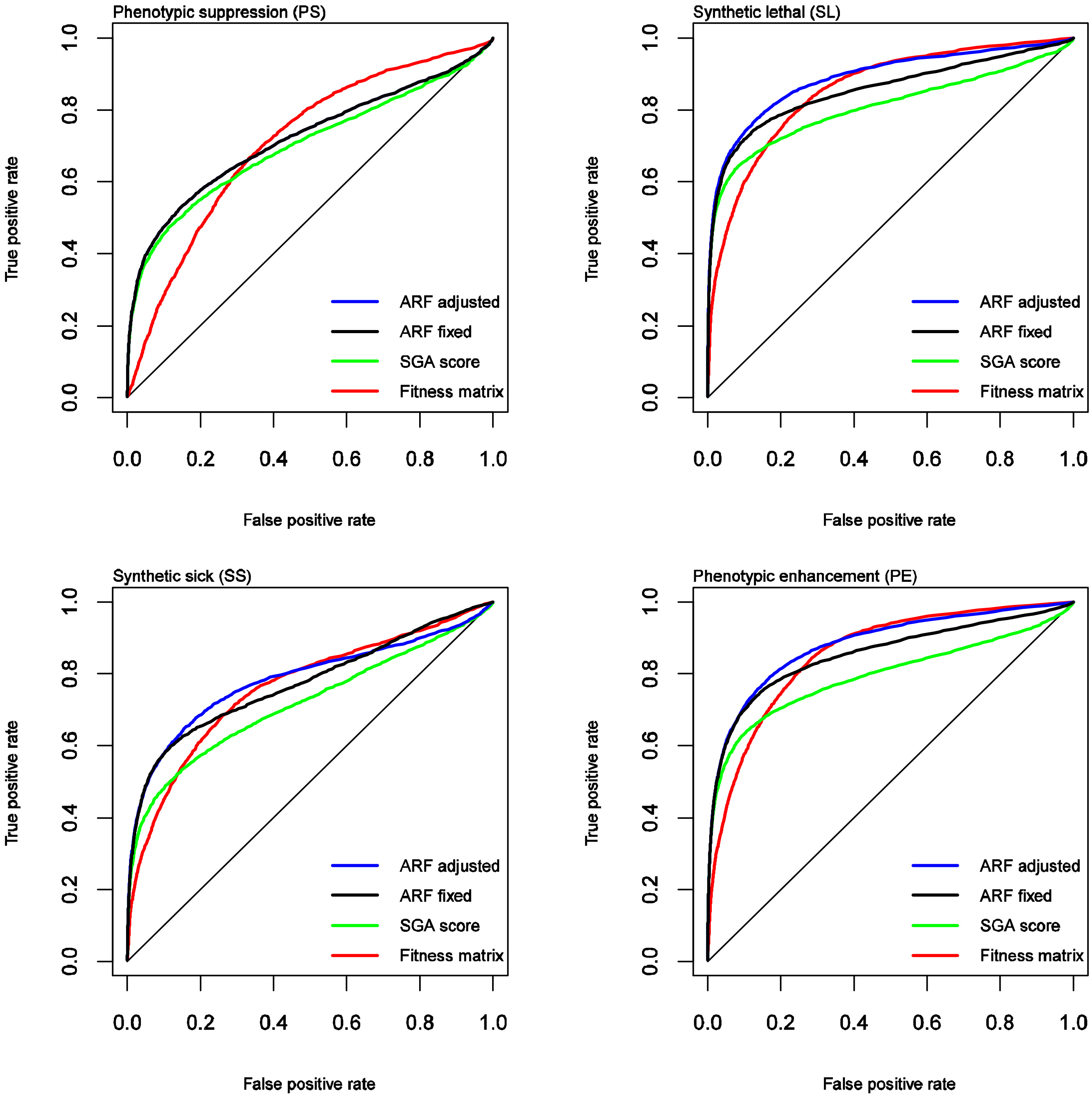

SGA-dataset: The ARF parameter fixed to the four interaction classes were t = 1.0, a = 0 (black curve). The ARF parameters adjusted to the positive (PS) and negative classes (SL, SS, and PE) were t = 1.0, a = 0 and t = 0.95, a = 1, respectively (blue curve). The original fitness measurements (red curve) and the customized SGA interaction scores (green curve) are shown as references. The product function was used for scoring the positive interactions and the minimum function for scoring the negative interactions. The values of the original double-mutant fitness matrix were traversed in reverse order when performing ROC-analyses in the PS category.
