# Supplementary material for: Genome-Wide Scoring of Positive and Negative Epistasis through Decomposition of Quantitative Genetic Interaction Fitness Matrices
Source: PLoS One. 2010 Jul 15;5(7):e11611. doi: 10.1371/journal.pone.0011611 (PMC2904709; doi:10.1371/journal.pone.0011611)
Supplement: Figure S2 — The full ROC curves showing the detection accuracy of the different genetic interaction categories in the GIM dataset using the QMA and ARF methods. The four interaction categories are shown as separate panels, and the two methods as separate sets of ROC curves on the two pages. (0.10 MB PDF) [file pone.0011611.s002.pdf]

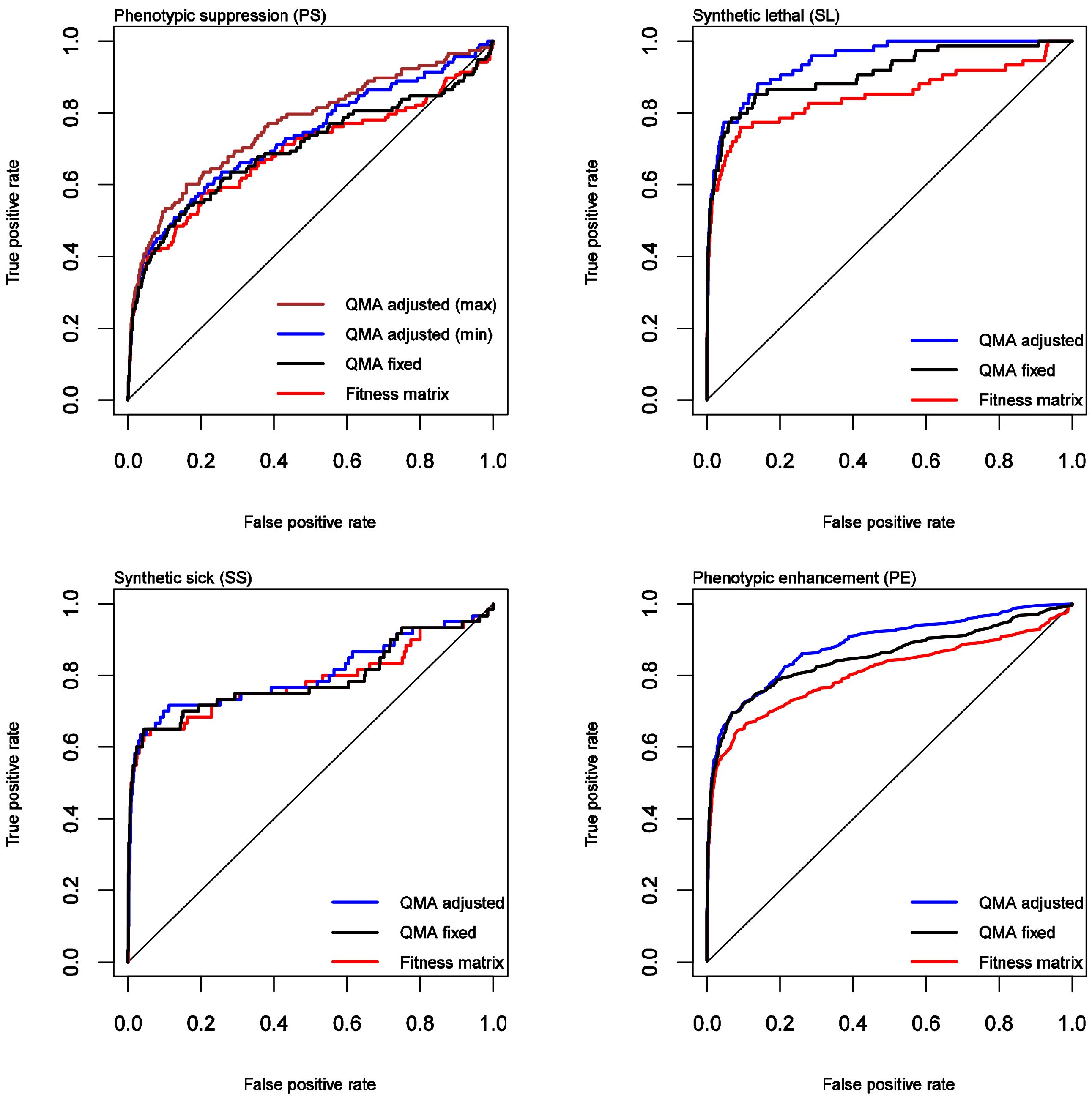

GIM-dataset: The QMA parameters fixed to the four interaction classes were p = 0.60, q = 0.50 (black curve). The QMA parameters adjusted to the positive (PS) and negative classes (SL, SS, and PE) were p = 0.15, q = 0.95 and p = 0.80, q = 0.25, respectively (blue curve). The original fitness measurements (red curve) are shown as reference. The minimum function was used for scoring the PS and SS categories, and the scaled epistatis function for scoring the SL and PE categories. In case the maximum scoring function was used in the PS category, then the QMA adjusted parameters were p = 0.05, q = 0.95 (brown curve).

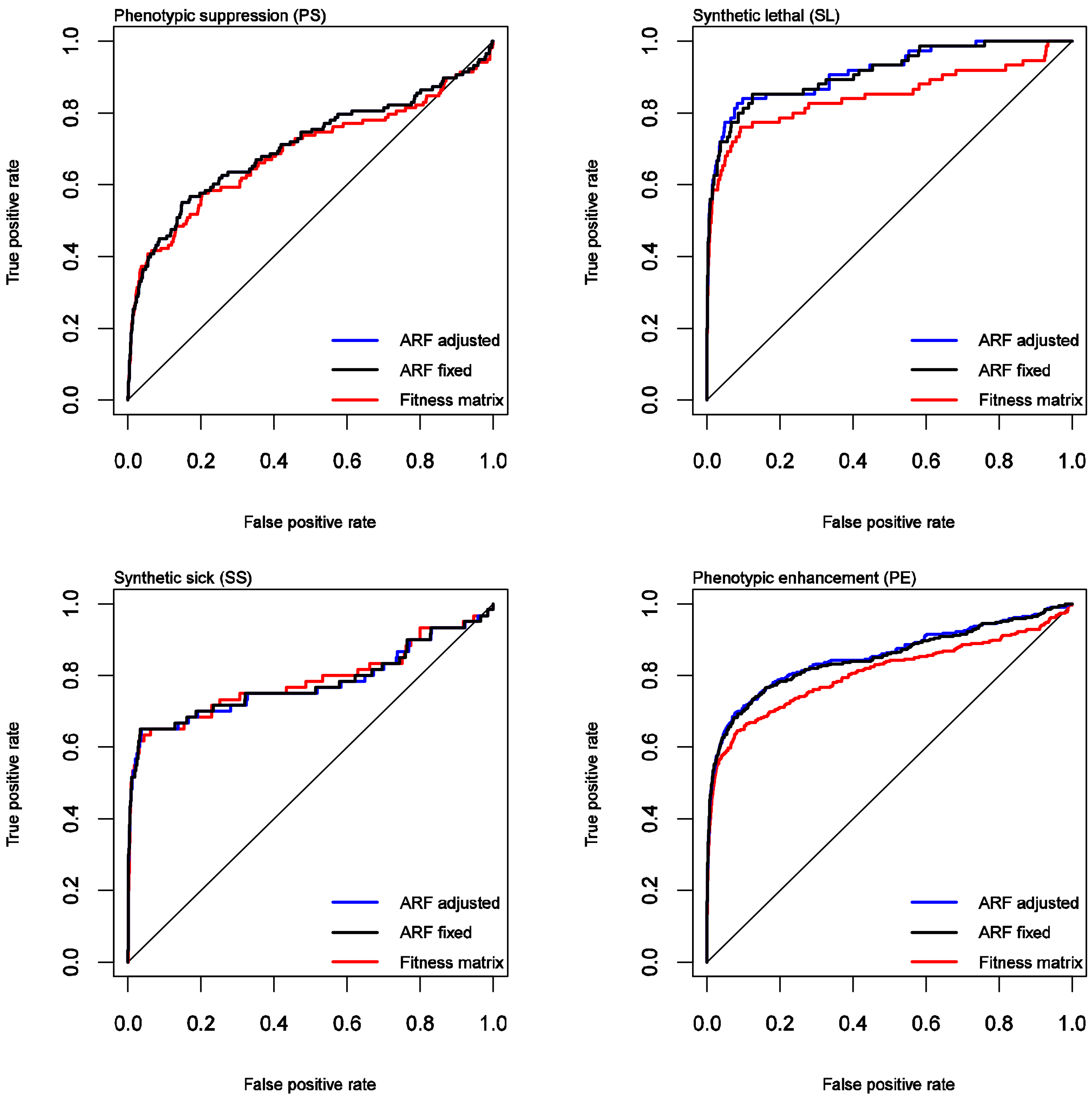

GIM-dataset: The ARF parameter fixed to the four interaction classes were *t* = 0.80, *a* = 0 (black curve). The ARF parameters adjusted to the positive (PS) and negative classes (SL, SS, and PE) were *t* = 0.80, *a* = 0 and *t* = 0.95, a = 0, respectively (blue curve). The original fitness measurements (red curve) are shown as reference. The minimum function was used for scoring the PS and SS categories, and the scaled epistatis function for scoring the SL and PE categories.
