# Supplementary material for: Genome-Wide Scoring of Positive and Negative Epistasis through Decomposition of Quantitative Genetic Interaction Fitness Matrices
Source: PLoS One. 2010 Jul 15;5(7):e11611. doi: 10.1371/journal.pone.0011611 (PMC2904709; doi:10.1371/journal.pone.0011611)
Supplement: Figure S3 — The full ROC curves showing the detection accuracy of the different genetic interaction categories in the E-MAP dataset using the QMA and ARF methods. The four interaction categories are shown as separate panels, and the two methods as separate sets of ROC curves on the two pages. (0.11 MB PDF) [file pone.0011611.s003.pdf]

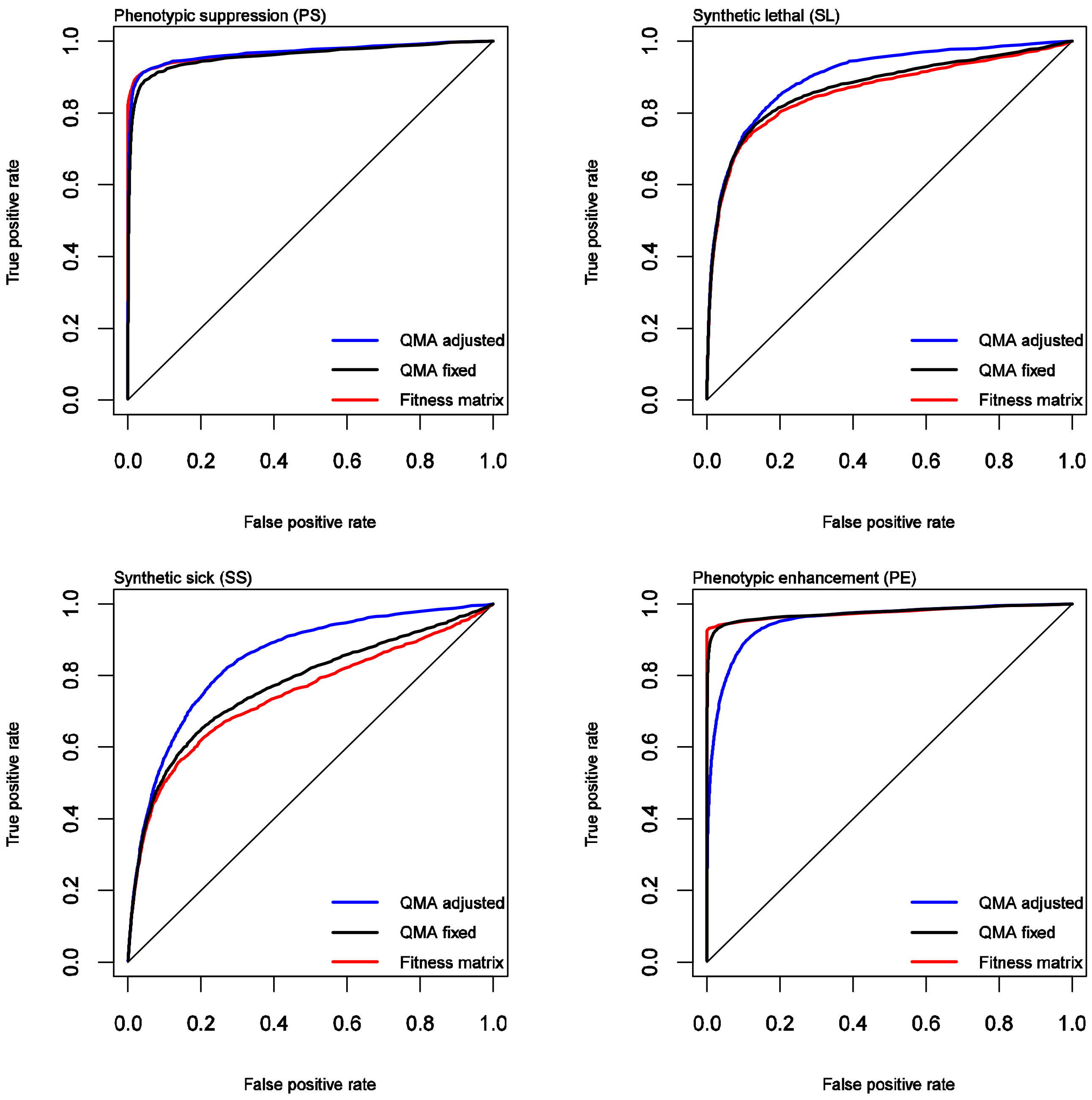

E-MAP-dataset: The QMA parameters fixed to the four interaction classes were p = 0.50, q = 0.60 (black curve). The QMA parameters adjusted to the positive (PS) and negative classes (SL, SS, and PE) were p = 0.30, q = 0.65 and p = 0.50, q = 0.15, respectively (blue curve). The original fitness measurements (red curve) are shown as reference. The minimum function was used for scoring each of the interaction categories. The dataset was pre-processed by subtracting the row means of the original interaction score matrix

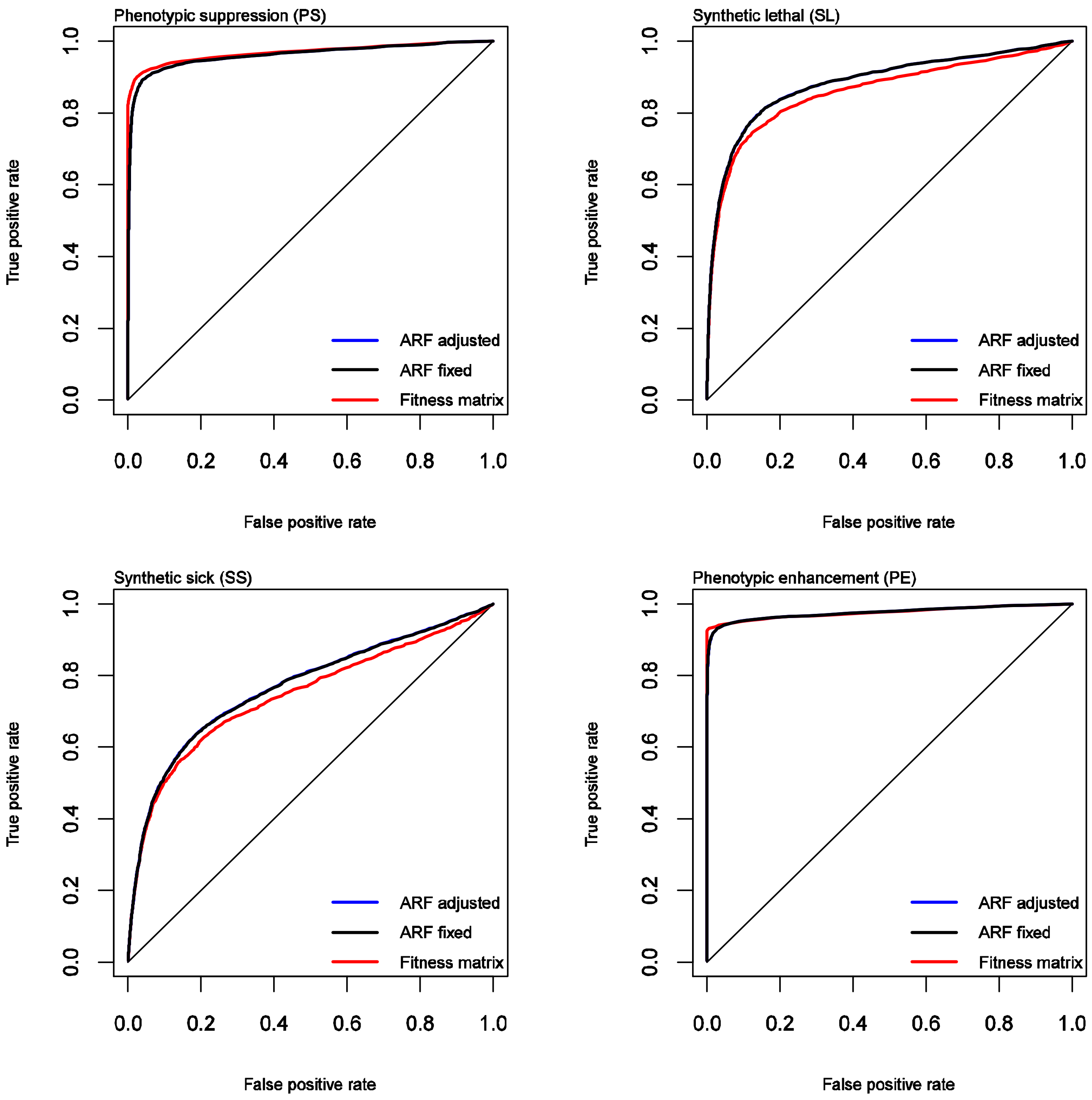

E-MAP-dataset: The ARF parameter fixed to the four interaction classes were t = 0.75, a = 0 (black curve). The ARF parameters adjusted to the positive (PS) and negative classes (SL, SS, and PE) were t = 0.75, a = 0 and t = 0.80, a = 0, respectively (blue curve overlaps almost entirely with the black curve). The original fitness measurements (red curve) are shown as reference. The minimum function was used for scoring all the other interaction categories, except the SL category, in which the product function was used. The dataset was pre-processed by subtracting the row means of the original interaction score matrix
