# Supplementary material for: Genome-Wide Scoring of Positive and Negative Epistasis through Decomposition of Quantitative Genetic Interaction Fitness Matrices
Source: PLoS One. 2010 Jul 15;5(7):e11611. doi: 10.1371/journal.pone.0011611 (PMC2904709; doi:10.1371/journal.pone.0011611)
Supplement: Table S1 — Candidate gene pairs showing evidence for positive genetic interactions as identified in the SGA dataset using the matrix decomposition strategy and the product scoring function (named ‘QMA score’). The nine ORF pairs already stored in the BioGRID-PS category are boldfaced. (0.01 MB PDF) [file pone.0011611.s004.pdf]

Query ORF	Array ORF	Fitness value	Fitness rank	QMA score	Adj rank	Fixed rank
YKL212W	YPL181W	1.17306	20396	0.68872	1	2
YPL079W	YBR200W	1.08076	448563	0.66770	2	4
YKL212W	YLR371W	1.02763	1085024	0.62263	3	9
YGR018C	YDR392W	1.30310	116	0.60235	4	1
YLR357W	YBR200W	0.72918	2811800	0.60163	5	6
YIL084C	YAL013W	1.41564	5	0.59673	6	10
YCR063W	YML128C	0.95029	1861654	0.59090	7	11
YNL085W	YBR200W	1.10760	222744	0.58252	8	72
YDL002C	YML128C	1.11447	180727	0.57679	9	15
YOL108C	YDR414C	1.05418	752482	0.57586	10	12
YGR125W	YIL040W	1.24194	1157	0.57216	11	13
YLR182W	YFL025C	0.73038	2808626	0.57054	12	23
YPL069C	YJL062W	1.13947	77095	0.57040	13	84
YLR357W	YDR392W	0.76261	2715739	0.56365	14	5
YLR357W	YOR058C	0.77375	2679267	0.56050	15	14
YLR357W	YBL031W	0.79904	2591334	0.55845	16	7
YPR097W	YDR126W	1.28910	193	0.55785	17	59
YDR094W	YBR200W	1.07647	493274	0.54806	19	157
YHR189W	YKL139W	0.97281	1676994	0.54598	20	29
**YPR141C**	**YMR198W**	0.98379	1574658	0.54578	21	124
YKR041W	YAL013W	1.55897	1	0.54295	22	3
YKR094C	YIL040W	1.26362	503	0.53960	23	8
YNR051C	YLR371W	1.21532	3384	0.53736	24	61
YDL100C	YML128C	1.20766	4701	0.53652	26	33
YLR357W	YPL036W	0.72866	2813147	0.53291	27	16
**YNL147W**	**YAL013W**	1.31296	82	0.52743	29	27
YKL212W	YLR170C	0.97139	1689509	0.52505	31	47
YOL004W	YJL124C	1.07651	492777	0.52206	32	56
YLR337C	YJL020C	0.76817	2697876	0.52105	33	40
YGL168W	YCR033W	1.04966	808461	0.51776	34	58
YLR357W	YIL040W	0.71788	2840032	0.51730	35	20
YNL071W	YOL081W	1.24369	1084	0.51666	36	60
YGL029W	YGL212W	0.89666	2187451	0.51641	37	28
YOR106W	YML128C	0.93063	1997388	0.51409	39	34
YLR337C	YDR072C	0.79051	2621722	0.51350	42	19
YLR357W	YHR167W	0.73917	2785239	0.51320	43	22
YOL108C	YDR162C	0.79094	2620187	0.51269	44	45
YOL108C	YJL183W	0.97395	1666736	0.51085	47	31
YDR123C	YAL013W	1.09328	330753	0.50976	49	30
**YDR289C**	**YOR026W**	1.00479	1354748	0.50903	50	70
YKL212W	YML128C	0.79821	2594267	0.50889	51	120
YKL212W	YPL174C	0.76657	2703074	0.50769	52	87
YDR245W	YJL183W	1.25059	836	0.50755	53	37
YKL212W	YDR414C	0.95251	1844834	0.50348	54	103
YLR357W	YOR304W	0.75691	2733469	0.50333	55	18
YIL040W	YAL013W	1.22287	2451	0.50311	56	53
YKL041W	YOR030W	0.90981	2119454	0.50078	58	97
YCR077C	YML128C	1.09067	353541	0.50069	59	139
YOL009C	YJL183W	0.88382	2248752	0.49894	61	82
YNL097C	YAL013W	1.31379	77	0.49747	62	38
YLR357W	YPR193C	0.78233	2650203	0.49746	63	21
YOL108C	YJR139C	0.80363	2574588	0.49557	65	55
YKL041W	YNR006W	0.83219	2465998	0.49327	66	65
YGR092W	YHR116W	0.97412	1665170	0.49326	67	26

YOL108C	YPR070W	0.81505	2532342	0.49147	68	78
**YDL100C**	**YGL020C**	1.33493	37	0.48971	69	62
YLR182W	YCR077C	0.62546	3011295	0.48942	70	148
YNR010W	YIL040W	1.08727	384648	0.48764	71	43
YMR198W	YIL040W	1.09542	312833	0.48706	72	79
**YMR078C**	**YHR191C**	1.13392	94162	0.48624	74	50
YNL079C	YGL020C	1.17656	17682	0.48543	76	112
YKL212W	YCL009C	0.93346	1979252	0.48098	80	107
**YGL020C**	**YER083C**	1.12069	148222	0.47937	81	44
YFL013C	YML128C	1.03304	1018000	0.47631	85	86
**YBR231C**	**YLR085C**	1.04698	842167	0.47584	86	153
YLR357W	YER073W	0.73686	2791480	0.47522	87	32
YOL108C	YMR299C	0.94439	1904512	0.47512	88	66
YHR075C	YKL139W	1.06147	663221	0.47483	89	85
**YLR337C**	**YJR073C**	0.63905	2991548	0.47471	90	67
YKL069W	YAL013W	1.02233	1150173	0.47460	91	143
YML097C	YDR108W	1.02802	1080186	0.47249	92	131
YDR485C	YMR198W	1.10106	268625	0.47175	93	90
YLR085C	YIL040W	1.08897	368905	0.47067	94	138
YLR357W	YDR162C	0.62546	3011293	0.47059	95	46
YPL047W	YAL013W	1.27912	281	0.46914	96	51
YML013W	YDL233W	1.10940	211108	0.46892	97	91
YML097C	YOL018C	0.93697	1956033	0.46764	98	105
YBR274W	YIL040W	1.30000	135	0.46596	99	42
YOR083W	YML128C	1.10712	225897	0.46316	102	76
YOL108C	YHR129C	0.82952	2476584	0.46237	105	110
YML012C-A	YLR371W	0.87492	2288857	0.46041	109	77
**YBR231C**	**YAL011W**	1.19351	8599	0.46003	110	108
YOR275C	YMR063W	1.08695	387610	0.45900	112	36
YOL108C	YER155C	0.86629	2326587	0.45897	113	64
YKR094C	YIL090W	0.81652	2526586	0.45598	117	106
YPL213W	YDR363W-A	0.82833	2481044	0.45591	119	146
YOL108C	YLL046C	0.93202	1988525	0.45458	122	113
YJL136C	YKL139W	0.91832	2071570	0.45284	127	122
YDL142C	YML128C	1.10040	273692	0.45172	128	68
YMR129W	YIL040W	1.37154	14	0.45146	129	24
YLR357W	YOL001W	0.62102	3017523	0.45068	132	49
YLR357W	YHR129C	0.65460	2966611	0.45026	134	71
YMR078C	YIL040W	1.04557	859961	0.44775	137	160
YGL173C	YKL139W	0.71734	2841348	0.44769	138	149
YOR275C	YOR030W	1.09354	328470	0.43841	152	94
YLR337C	YDR297W	0.68437	2912735	0.43670	155	156
YBR103W	YML128C	1.14674	58617	0.43610	156	92
